# Supplementary material for: Water stable molecular n-doping produces organic electrochemical transistors with high transconductance and record stability
Source: Nat Commun. 2020 Jun 12;11:3004. doi: 10.1038/s41467-020-16648-0 (PMC7293298; doi:10.1038/s41467-020-16648-0)
Supplement: Supplementary file 1 — Supplementary Information [file 41467_2020_16648_MOESM1_ESM.pdf]

Supplementary Information
for

Water Stable Molecular N-Doping Produces Organic Electrochemical Transistors with High Transconductance and Record Stability

by Paterson et al

Supplementary Figures

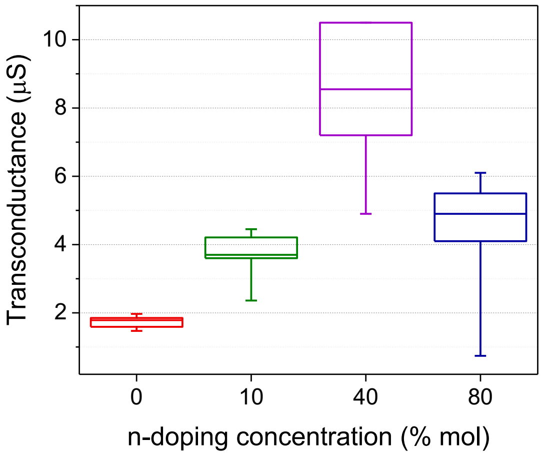

Supplementary Figure 1. Organic electrochemical transistor (OECT) statistics. Statistical variation in the maximum transconductance, g_{m_max}, where g_{m_max} is taken at V_G ≈0.5 V over six organic electrochemical transistors for each system.

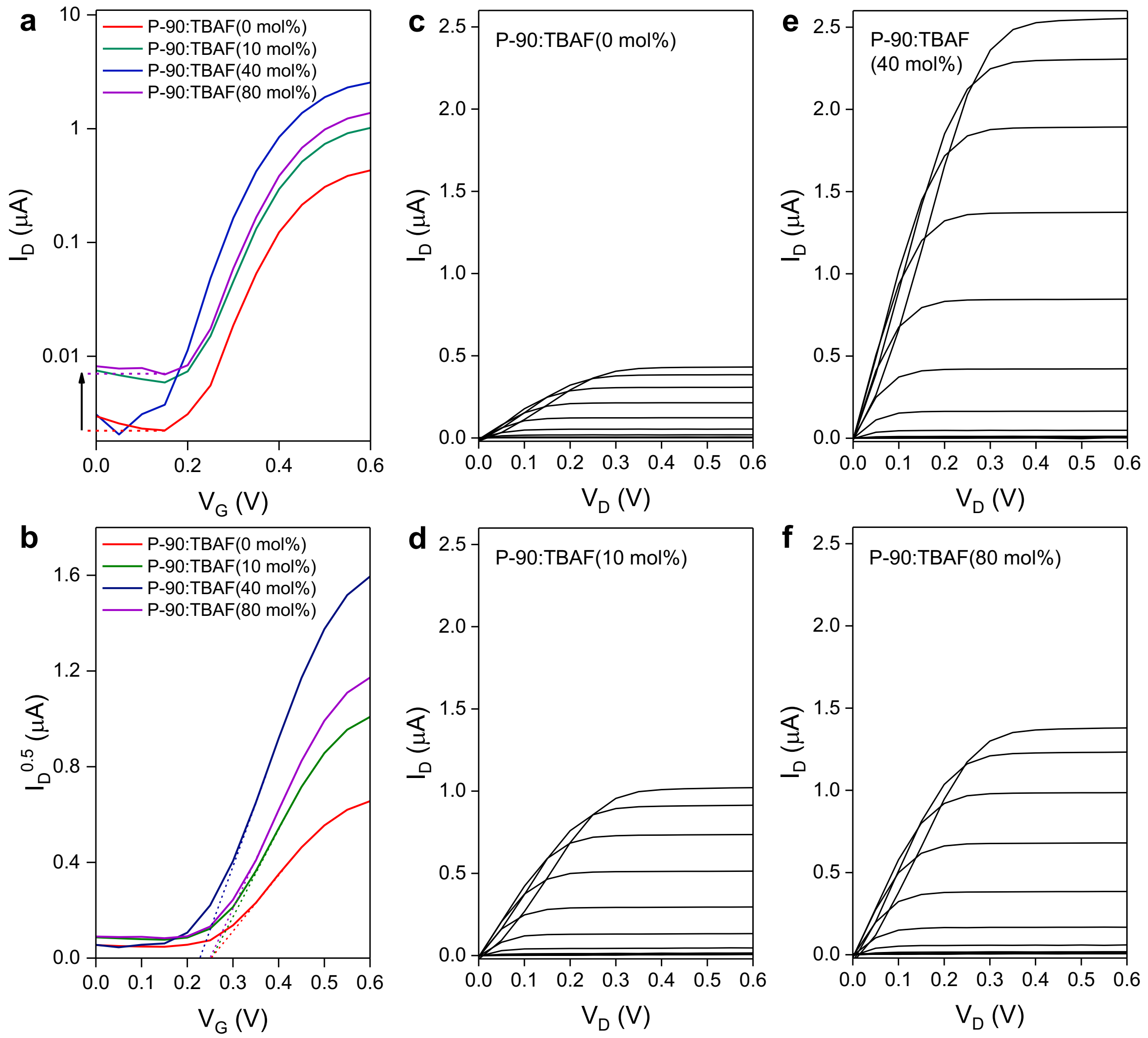

Supplementary Figure 2. N-doped organic electrochemical transistors. Comparative (a) transfer characteristics and (b) square root of I_D for P-90 OECTs with 0, 10, 40 and 80 mol% TBAF. Output characteristics for: (c) P-90:TBAF(0 mol%), (d) P-90:TBAF(10 mol%), (e) P-90:TBAF(40 mol%) and (f) P-90:TBAF(80 mol%) OECTs.

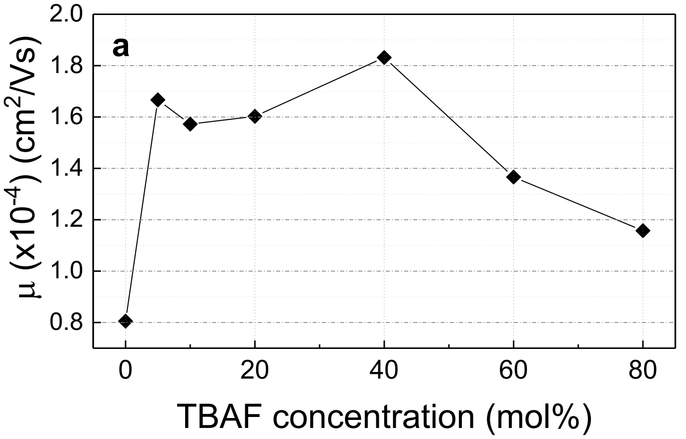

Supplementary Figure 3. Organic electrochemical transistor (OECT) charge carrier mobility. P-90 electron mobility (μ) for OECTs containing 0, 5, 10, 20, 40, 60 and 80 mol% TBAF, showing that TBAF increases μ, with a maximum value at 40 mol% decreasing with higher concentrations of TBAF. μ is determined by measuring the transit time of the electronic carriers using an impedance matching method.

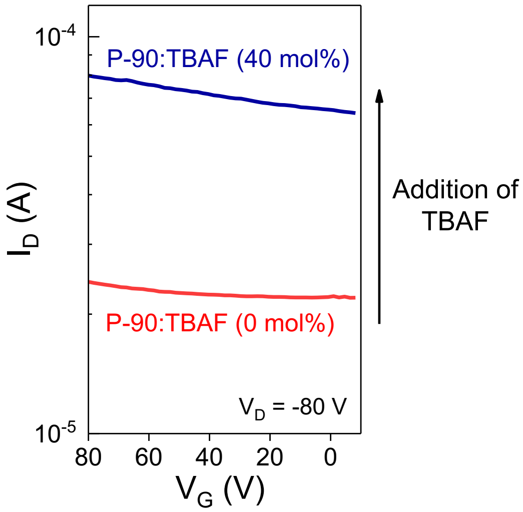

Supplementary Figure 4. Organic field-effect transistor (OFET) measurements. OFET measurements on P-90:TBAF(0 mol%) and P-90:TBAF(40 mol%), showing that, despite the lack of field-effect induced behaviour, the introduction of 40 mol% TBAF has increased the P-90 conductivity. V_G is varied from 0 to 80 V at a fixed 80 V between the source and drain.

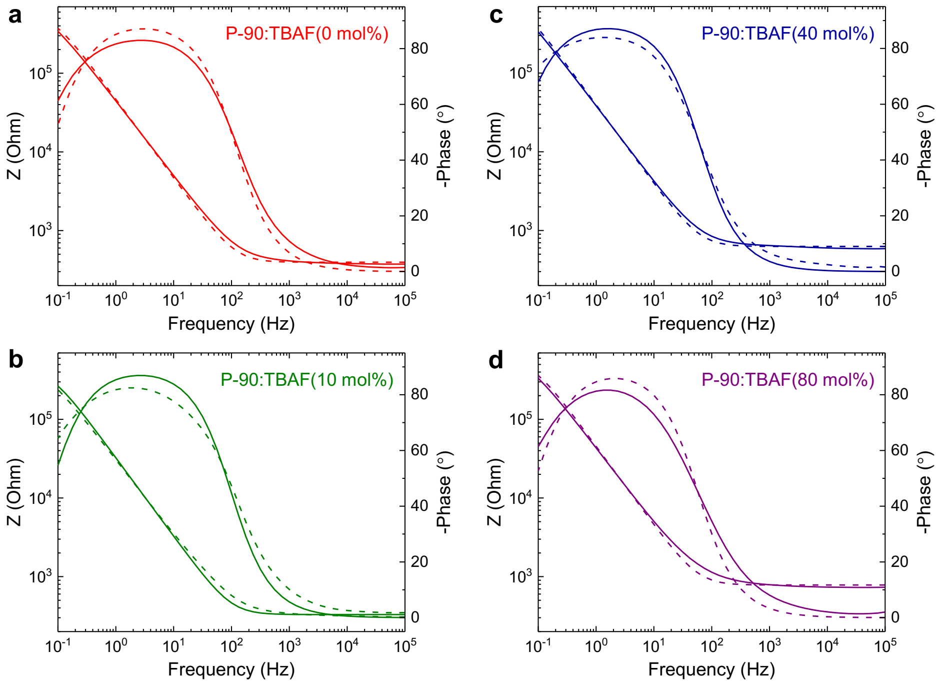

Supplementary Figure 5. Electrochemical impedance spectroscopy (EIS). Frequency dependent impedance and phase data (solid line) and the plots extracted from the equivalent R(RC) circuit model (dashed line) for (a) P-90:TBAF(0 mol%), (b) P-90:TBAF(10 mol%), (c) P-90:TBAF(40 mol%) and (d) P-90:TBAF(80 mol%). Each system is measured at a doping potential of 0.5 V vs Ag/AgCl in 0.1 M $NaCl_{(aq)}$ electrolyte. All films were deposited onto 580 x 580 μm Au electrodes. Corresponding film thicknesses are given in **Table 1**.

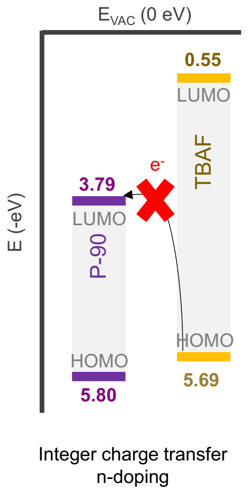

Supplementary Figure 6. Density Functional Theory: integer charge transfer. Energy level diagram indicating that n-doping via integer charge transfer from TBAF to P-90 is not possible.

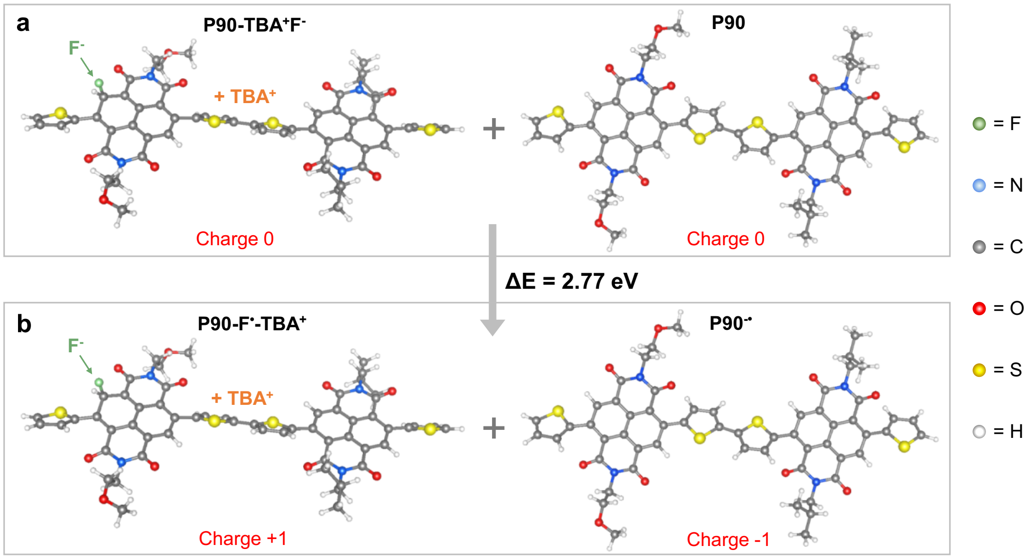

Supplementary Figure 7. Density Functional Theory: TBA^+ cation location. The same n-doping mechanisms described in Figure 2b-e, with a TBA^+ cation next to the fluorinated site in P-90.

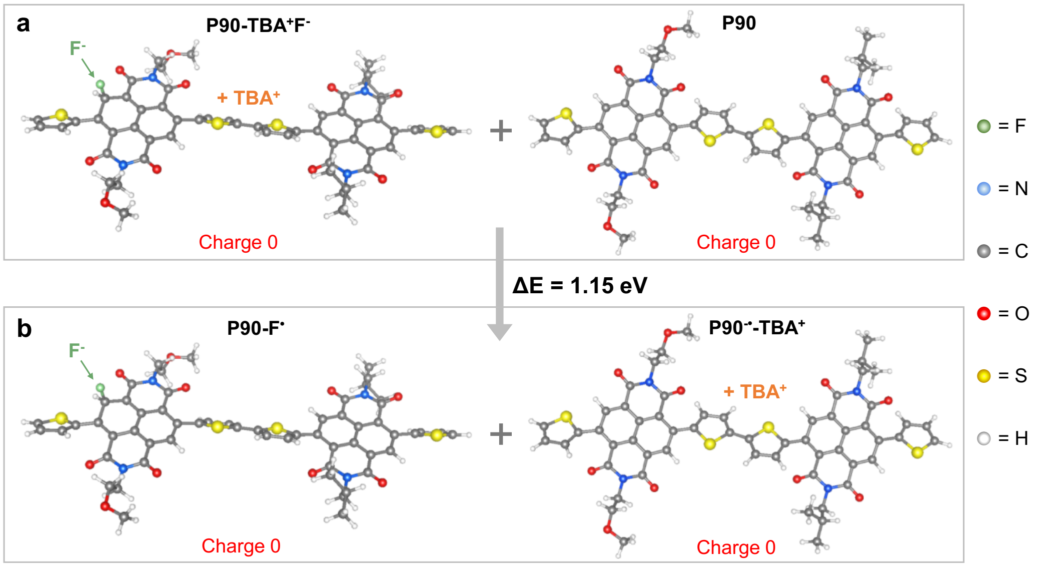

Supplementary Figure 8. Density Functional Theory: TBA^+ cation location. The same n-doping mechanisms described in Figure 2b-e, with a TBA^+ cation that moves with the released electron.

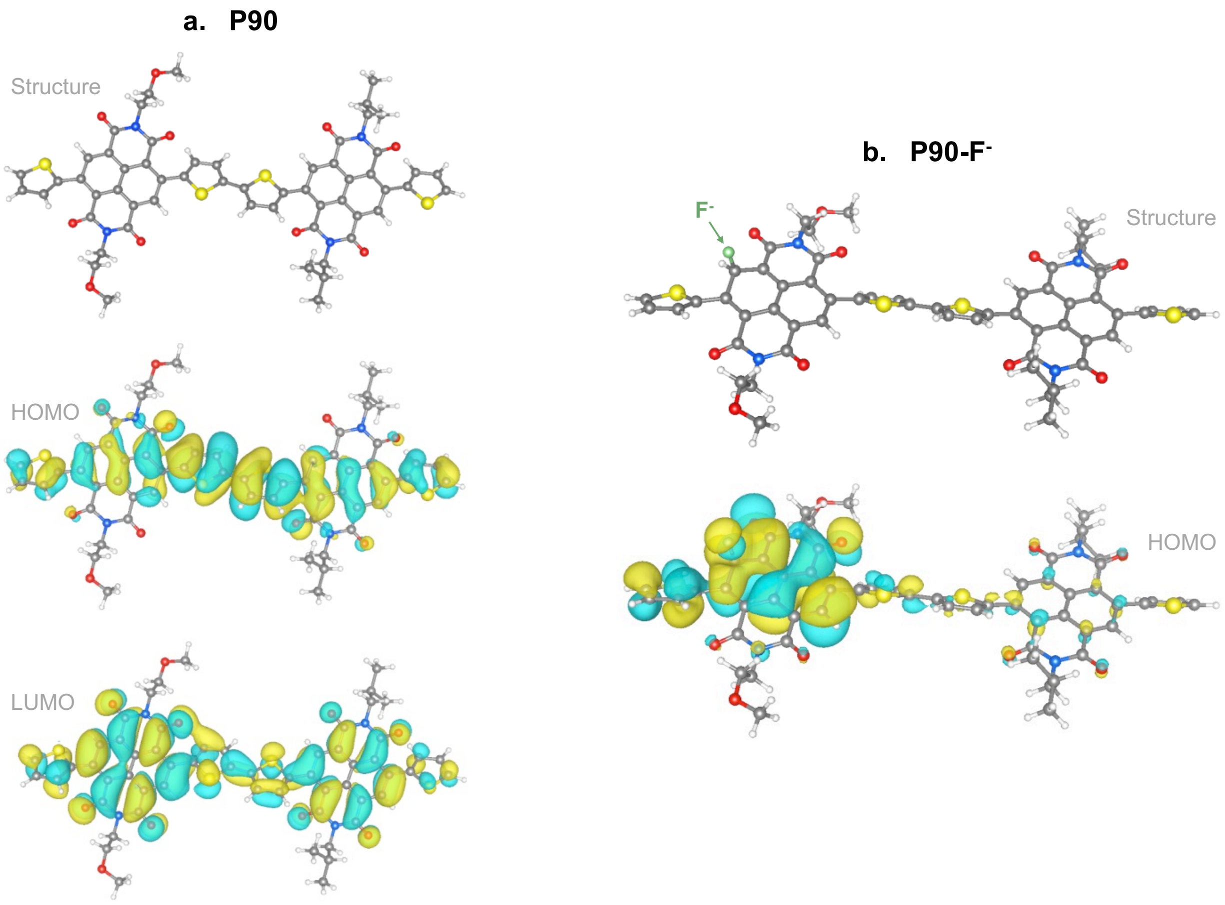

Supplementary Figure 9. Density Functional Theory: highest occupied molecular orbitals. (a) Structure, HOMO and LUMO of a P-90 monomer. (b) Structure and HOMO of a P-90 complex, where an F atom has been transferred from a TBAF molecule to a P-90 monomer. The LUMO of the pristine P-90 and HOMO of the P-90 complex show a higher degree of electron delocalisation on the NDI unit(s).

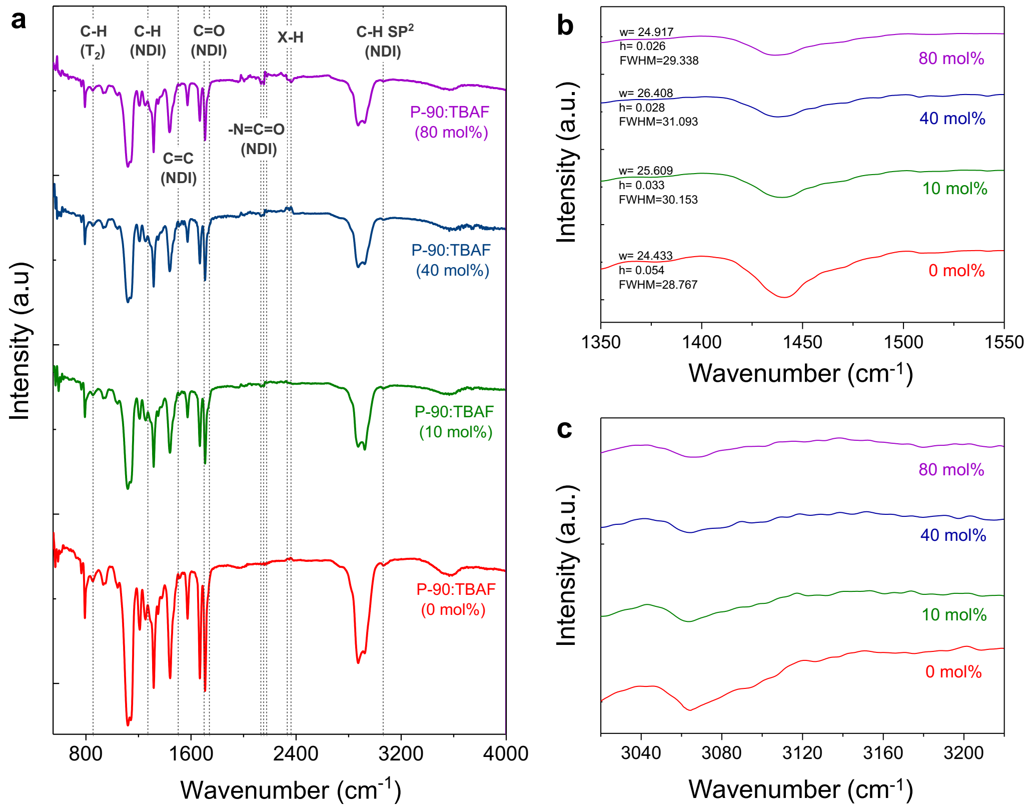

Supplementary Figure 10. Fourier-transform infrared (FTIR) spectroscopy. (a) Overview of Fourier-transform infrared (FTIR) spectroscopy results. (b) Stretching of C=C aromatic in NDI; we note that, since there is no shift in the T2 unit C-H vibration, electron delocalisation only occurs on the NDI unit, and not on the T2 unit. (c) SP^2 C-H stretching in aromatic ring of NDI.

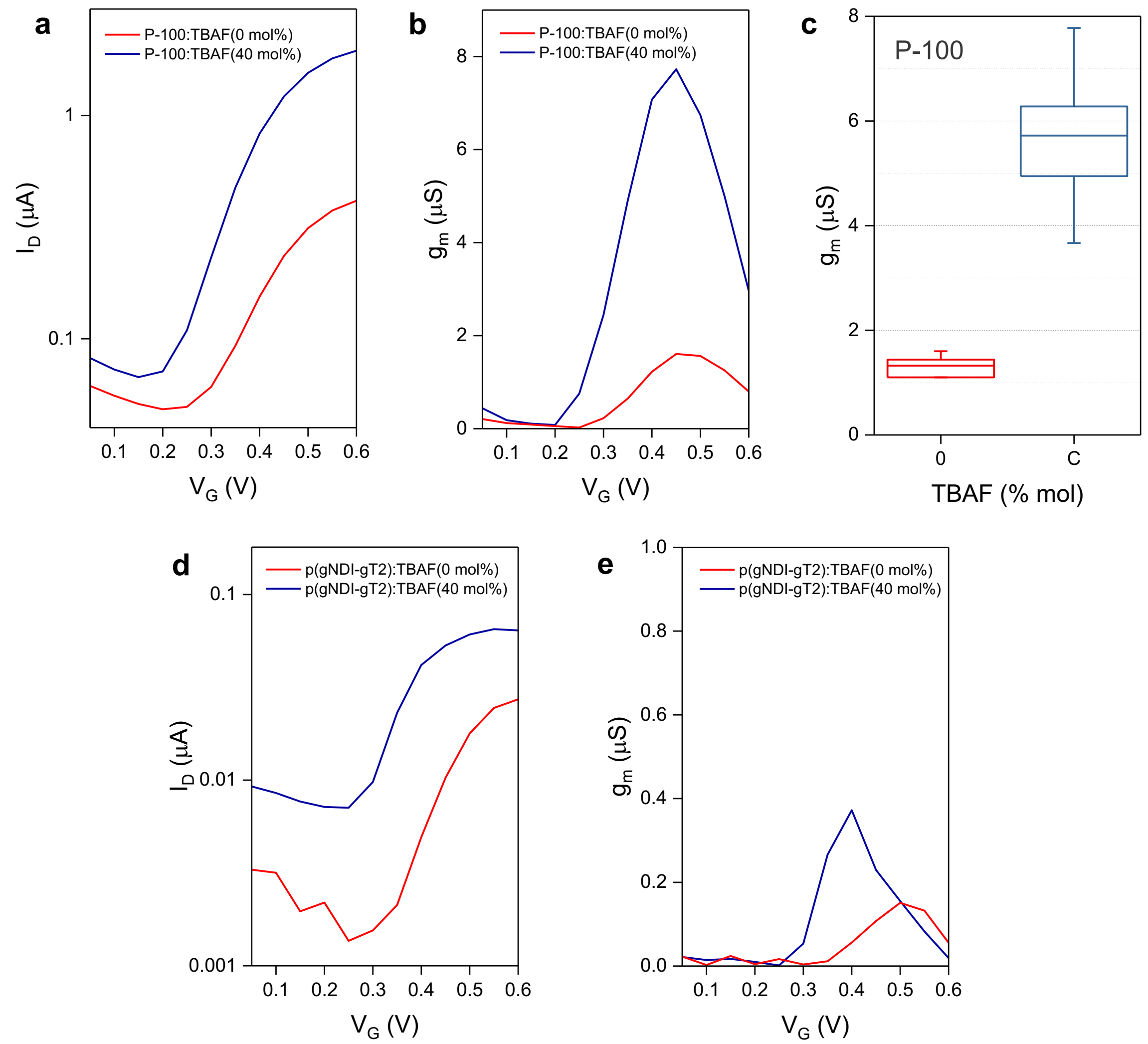

Supplementary Figure 11. N-doped organic electrochemical transistors. (a) Transfer curve, (b) maximum transconductance and (c) transconductance statistics over 6 P-100:TBAF OECTs with 0 mol% and 40 mol% TBAF. Data has not been normalised to film thicknesses, which are 88 nm and 50 nm for P 100:TBAF(0 mol%) and P 100:TBAF(40 mol%), respectively. (d) Transfer curve and (e) maximum transconductance of best-performing p(gNDI-gT2) devices containing 0 mol% and 40 mol% TBAF. Data has not been normalised to film thickness, which are 59 nm and 55 nm for p(gNDI-gT2) devices containing 0 mol% and 40 mol% TBAF, respectively. OECT statistics are unavailable for p(gNDI-gT2) due to poor device performance and uniformity. All thicknesses were measured using a mechanical profilometer.

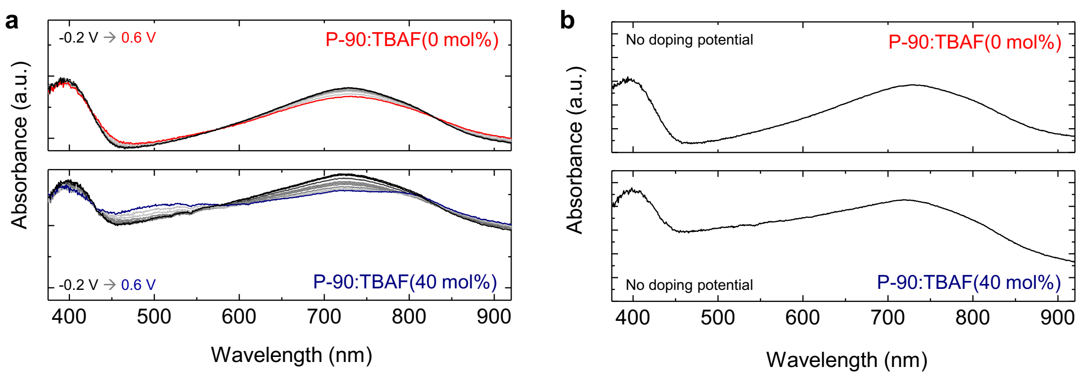

Supplementary Figure 12. Spectroelectrochemistry. (a) Raw absorbance spectra during electrochemical charging in 0.1 M $NaCl_{(aq.)}$. (b) Initial absorbance spectra, prior to any voltage application.

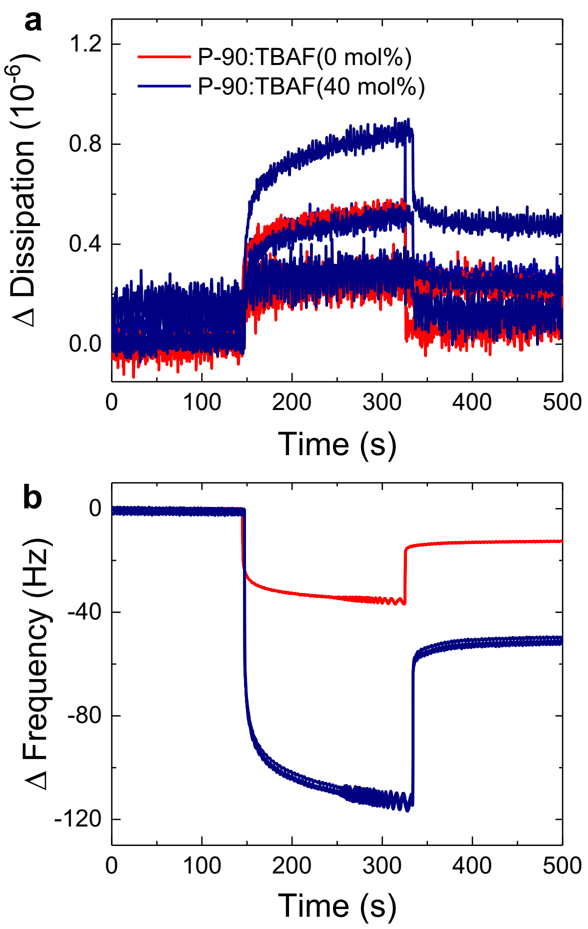

Supplementary Figure 13. Electrochemical quartz crystal microbalance with dissipation (EQCM-D). Change in (a) energy dissipation (b) frequency upon application of a doping pulse at -0.45 V vs. Ag/AgCl in 0.1 M $NaCl_{(aq.)}$. The dissipation data are shown for 3 overtones. The data were used to extract the mass values in Figure 3c. The fact that the frequency shifts change drastically with voltage for the P-90:TBAF(40 mol%) compared to P-90:TBAF(0

mol%), whist the energy dissipation for both systems in (a) remains almost the same, indicates that penetrating ions lead to smaller changes in the mechanical properties of the former.

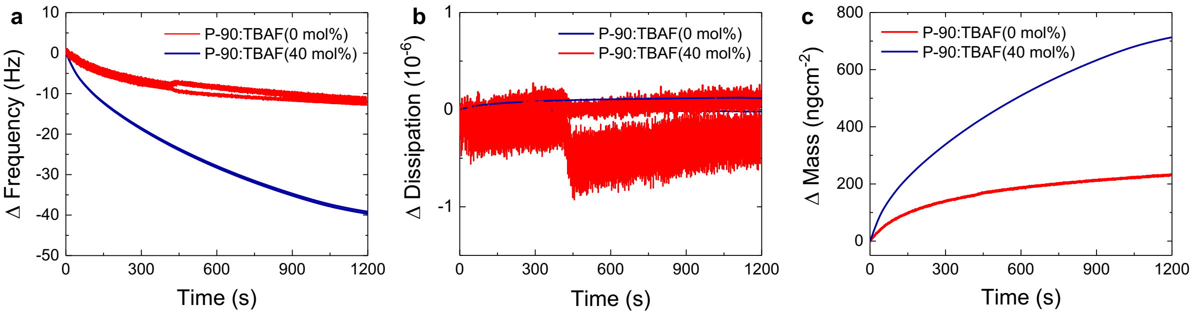

Supplementary Figure 14. Passive diffusion of water and ions. Electrochemical quartz crystal microbalance with dissipation (EQCM-D) monitoring data showing change in (a) frequency, (b) energy dissipation and (c) mass of the P-90:TBAF(0 mol%) and P-90:TBAF(40 mol%) thin-films immersed into $NaCl_{(aq.)}$ 0.1M electrolyte, without applying any bias. The P-90:TBAF(40 mol%) exhibits significantly greater, "passive" mass uptake compared to P-90:TBAF(0 mol%).

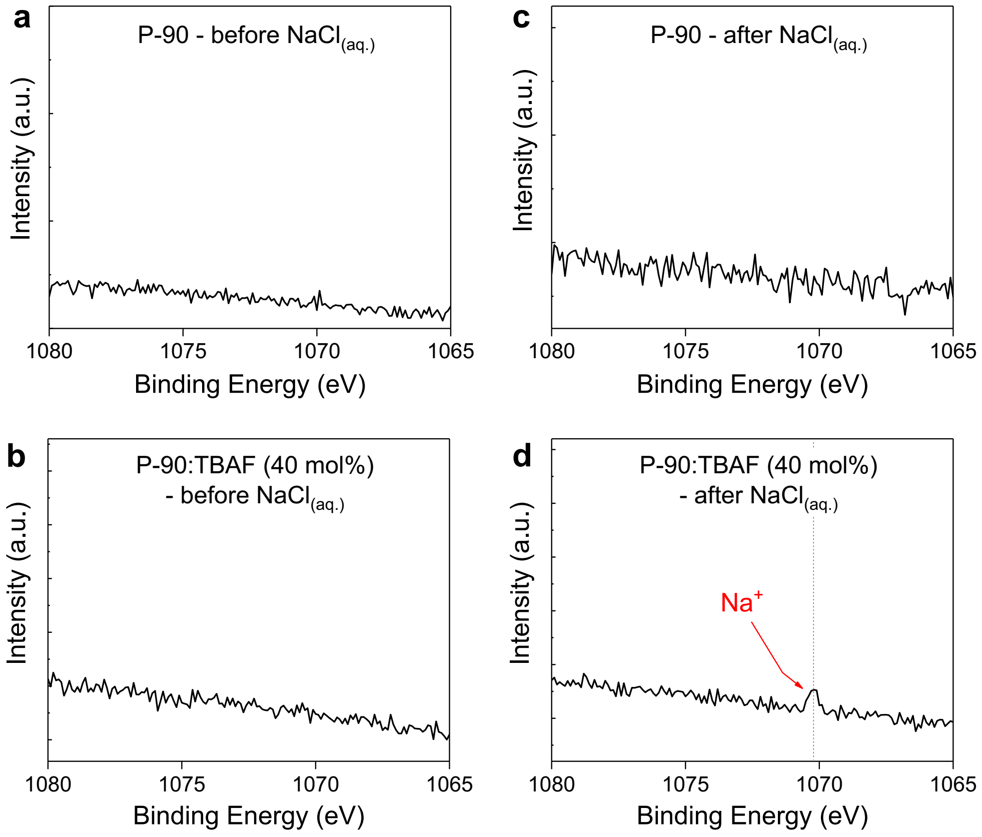

Supplementary Figure 15. X-ray photoelectron spectroscopy (XPS) showing the impact of TBAF on electrolyte cations without applied voltage. (a) Pristine P-90:TBAF(0 mol%) and (b) best-performing P-90:TBAF(40 mol%) prior to submersion in 0.1 M $NaCl_{(aq.)}$ electrolyte. (c) Pristine P-90:TBAF(0 mol%) and (d) best-performing P-

90:TBAF(40 mol%) after overnight submersion in 0.1 M $NaCl_{(aq.)}$ electrolyte. The data shows that the presence of TBAF in the film draws Na^+ cations into the film in the absence of an external applied voltage (such as V_G)[1,2].

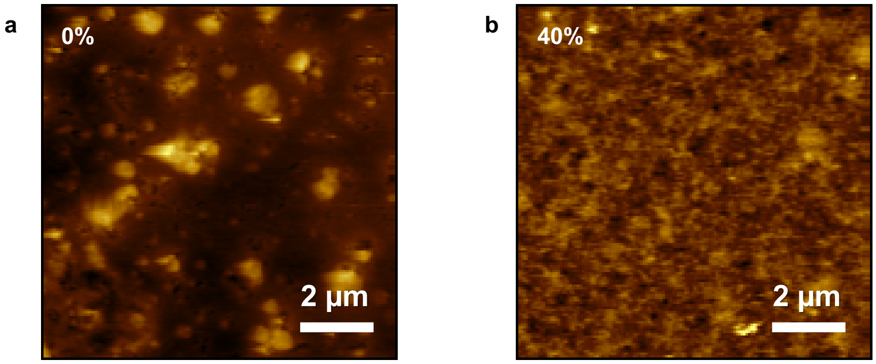

Supplementary Figure 16. Atomic force microscopy. Large area topography images of (a) P-90:TBAF(0 mol%) and (b) P-90:TBAF(40 mol%) thin-films. Scale bars are 2 µm. All films were deposited onto 580 x 580 µm Au electrodes and had been submerged in electrolyte prior to measurement.

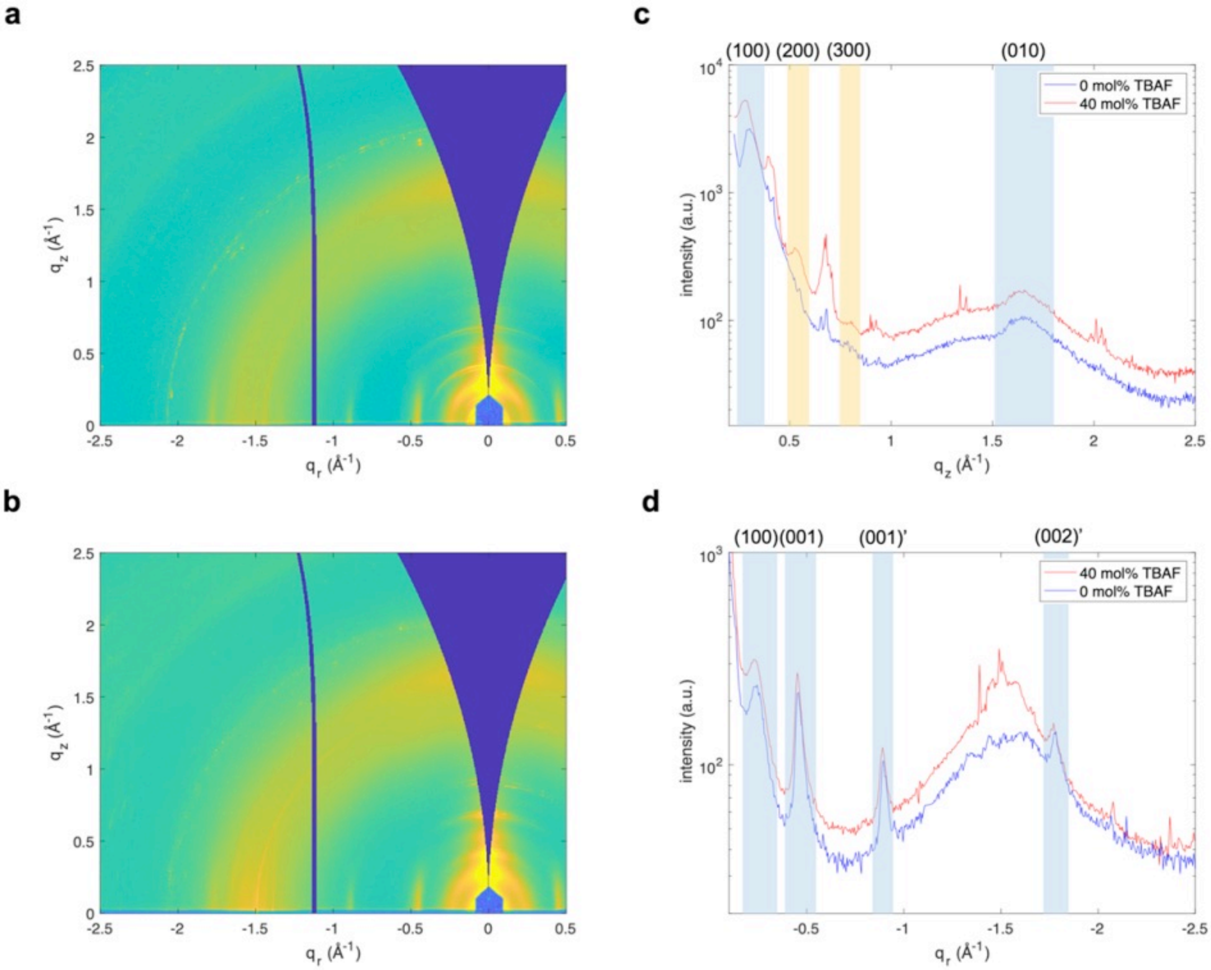

Supplementary Figure 17. Grazing Incidence Wide Angle Scattering. 2-D scattering patterns of (a) pristine P-90:TBAF(0 mol%) and (b) best-performing P-90:TBAF(40 mol%), with (c) in-plane and (d) out-of-plane line cuts of

each. Due to small sample size edge effects, there is significant edge-lip/particulate scattering present as sharp semi-isotropic scattering most prominent in the out-of-plane scatter. Characteristic thin-film scattering peaks are still clearly discernible and highlighted in blue. Higher order out-of-plane lamellar peaks, highlighted in yellow, appear marginally stronger in the P-90:TBAF(40 mol%), though the relatively small magnitude of the effect combined with the conflation of edge-lip/particulate scattering limits quantification of this change.

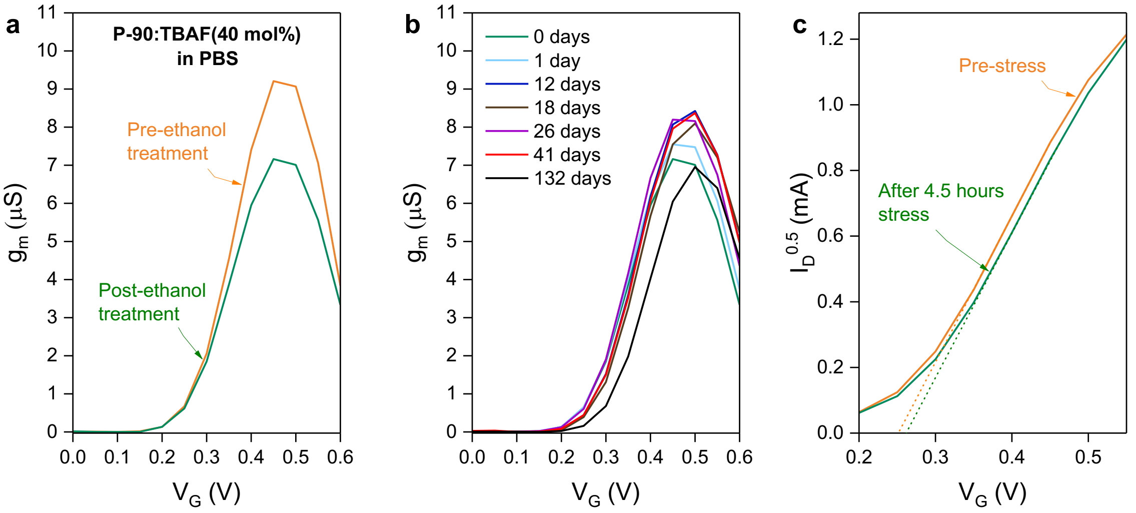

Supplementary Figure 18. N-doped organic electrochemical transistor stability. (a) Transconductance of P-90:TBAF(40 mol%) OECTs measured in phosphate-buffered saline (PBS) before and after a 30 minute sterilisation treatment in ethanol. (b) Transconductance data for OECTs aged in PBS for up to 132 days. (c) Square root of I_D for pre- and post- bias-stress stability data, showing a slight shift in V_T after 4.5 hours of pulsed bias-stressing, at 10 second intervals, at $V_G = V_D = 0.4$ V.

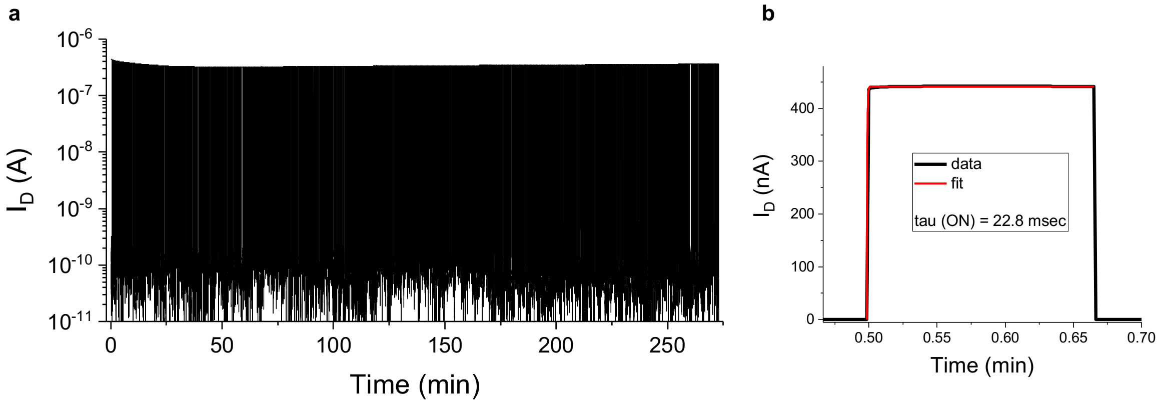

Supplementary Figure 19. N-doped organic electrochemical transistor operational stability. (a) Drain current as a function of time in operational stability tests, for best-performing P-90:TBAF(40 mol%) OECTs. Gate voltage pulses were applied for 10 seconds with an interval of 10 seconds between successive pulses while the drain voltage was kept constant ($V_G = V_D = 0.4$ V). (b) An exponential fit (red line) with a time constant of 22.8 msec for the turn ON transient (black line).

Supplementary Tables

Supplementary Table 1. N-type organic electrochemical transistor performance. Comparison of the relevant n-type organic electrochemical transistor transconductance figures of merit and stability, as reported in this work and in the literature.

N-type material	Electrochemical transistor figures of merit								
	L	W	d	g_{m_max} (μS)	Peak current (mA)	Stability	Response time (ON)	$\frac{gm_{max}.L}{W.d}$ ($S.cm^{-1}$)	$\frac{\text{Peak current}}{W.d.L}$ ($A.\mu m^{-3}$)
C60-TEG[8]	20 μm	400 μm	140 nm	4.1 (V_G = 0.8V)	≈ 0.06 (V_G = 0.8V; V_D = 0.6V)	15% current degradation after 5 min repeated cycling at V_G = 0.5 V (V_{th} = 0.55V)	80 ± 10 ms	0.015	0.053
BBL[9]	20 μm	39 mm	180 nm	9700 (V_G = 0.7V)	≈ 3 (V_G =0.7V; V_D =0.6V)	No current degradation after 1 h successive 5 s V_G-pulses at 0.5 V.<br>No g_m degradation after 3 months ambient storage.	0.9 s	0.276	0.021
p(gNDI-gT2)[10]	10 μm	100 μm	200 nm	21.7 (V_G = 0.5V)	0.00385 (V_G = 0.6V; V_D = 0.6V)	No current degradation after 2 h successive 5 s V_G-pulses at 0.5 V	5 ms	0.108	0.019
P-90 (undoped)[11*]	10 μm	100 μm	135 nm	0.8 (V_G = 0.5V)	0.00019 (V_G = 0.6V; V_D = 0.6V)	-	-	0.006	0.001
This work	10 μm	100 μm	116 nm	10.5 (V_G = 0.5V)	0.00255 (V_G = 0.6V; V_D = 0.6V)	No current or g_m degradation after 4.5 h successive 10 s V_G-pulses at 0.5 V<br><br>ca. 20% decrease in g_m after ethanol-sterilisation<br><br>After more than 4 months storage in PBS, g_m decreases by only ≈3%	24 ± 2 ms	0.091	0.022
	10 μm	100 μm	50 nm	7.7 (P-100) (V_G = 0.45V)	≈0.00195 (V_G = 0.6V; V_D = 0.5V)	-	-	0.154 (P-100)	0.039 (P-100)

*The first report of the polymer has a normalized g_{m_max} of 0.021 S/cm.[12] We report here the data from our previous studies to eliminate batch-to-batch differences.

Supplementary Table 2. Fourier-transform infrared (FTIR) spectroscopy. Summary of vibrational spectra and physical explanations.

	TBAF content in P-90 (mol%)				Physical description
	0	10	40	80	
Vibrational bands	1708 [3,4]	1707	1706	1706	C=O stretching in NDI (symmetric)
	1668 [3,4]	1667	1666	1666	C=O stretching in NDI (antisymmetric)
	792 [3,4]	792	792	792	C-H out-of-plane bending vibration in T2
	1442 [5]	1439	1437	1436	C=C aromatic stretching in NDI
	3065 [6]	3062	3062	3062	SP^2 C-H stretching in NDI aromatic ring
	1209	1207	1207	1206	C-H in-plane bending vibration in NDI aromatic ring
	-	-	-	2335<br>2363 [7]	X-H heteroatom hydride vibrations
	-	2175<br>2154<br>2133 [7]	2175<br>2154<br>2133	2175<br>2154<br>2133	-N=C=O asymmetrical stretching in NDI ring unit

Supplementary References

1. Morgan, W. E., Van Wazer J. R., Stec, W. J. Inner-orbital photoelectron spectroscopy of the alkali metal halides, perchlorates, phosphates, and pyrophosphates. *J. Am. Chem. Soc.* **95**, 751-755 (1973).

2. Wagner, C. D. Chemical shifts of Auger lines, and the Auger parameter. *Faraday Discuss. Chem. Soc.* **60**, 291-300 (1975).

3. Giussani, E., Fazzi, D., Brambilla, L., Caironi, M., Castiglioni, C. Molecular Level Investigation of the Film Structure of a High Electron Mobility Copolymer via Vibrational Spectroscopy. Macromolecules **46**, 2658-2670 (2013).

4. Anton, A. M., Steyrleuthner, R., Kossack, W., Neher, D., Kremer, F. Infrared Transition Moment Orientational Analysis on the Structural Organization of the Distinct Molecular Subunits in Thin Layers of a High Mobility n-Type Copolymer. *J. Am. Chem. Soc.* **137**, 6034-6043 (2015).

5. Mani, T., Grills, D. C., Newton, M.D., Miller, J.R. Electron Localization of Anions Probed by Nitrile Vibrations. *J. Am. Chem. Soc.* **137**, 10979-10991 (2015).

6. Guha, S., Goodson, F. S., Roy, S., Corson, L. J., Gravenmier, C. A, Saha, S. Electronically Regulated Thermally and Light-Gated Electron Transfer from Anions to Naphthalenediimides. *J. Am. Chem. Soc.* **133**, 15256-15259 (2011).

7. Coates, J. Interpretation of Infrared Spectra, A Practical Approach in *Encyclopedia of Analytical Chemistry: Applications, Theory and Instrumentation* (2006).

8. Bischak, C. G., Flagg, L. Q., Yan, K., Li, C.-Z., Ginger, D. S. Fullerene Active Layers for n-Type Organic Electrochemical Transistors. *ACS Appl. Mater. Interfaces* **11**, 28138-28144 (2019).

9. Sun, H. *et al.* Complementary Logic Circuits Based on High-Performance n-Type Organic Electrochemical Transistors. *Adv. Mater.* **30**, 1704916 (2018).

10. Giovannitti, A. *et al.* N-type organic electrochemical transistors with stability in water. *Nat. Commun.* **7**, 13066 (2016).

11. Paterson, A. F. *et al.* On the Role of Contact Resistance and Electrode Modification in Organic Electrochemical Transistors. *Adv. Mater.* **31**, 1902291 (2019).

12. Giovannitti, A. *et al.* The Role of the Side Chain on the Performance of N-type Conjugated Polymers in Aqueous Electrolytes. *Chem. Mater.* **30**, 2945-2953 (2018).
